# High-Dose Dexmedetomidine for Severe Hyperactive Delirium Secondary to Intravenous Levetiracetam on Two Separate Occasions in the Same Patient

**DOI:** 10.1155/2022/1843774

**Published:** 2022-07-02

**Authors:** Chad Ward, Kiran Khalid, Nicole Rozette

**Affiliations:** ^1^Department of Pediatric Intensive Care Medicine, Carilion Children's Hospital, Virginia Tech Carilion School of Medicine, Roanoke, VA, USA; ^2^Department of Psychiatry and Behavioral Medicine, Virginia Tech Carilion School of Medicine, Roanoke, VA, USA; ^3^Department of Pharmacy Services, Carilion Roanoke Memorial Hospital, Roanoke, VA, USA

## Abstract

We describe the case of a 5-year-old male who developed severe hyperactive delirium with aggressive violent behavior following the administration of IV levetiracetam for the treatment of status epilepticus on two occasions. The child's symptoms ranged from attacking his parents and the intensive care staff. Risperidone was given without any improvement in symptoms. A high-dose continuous infusion of IV dexmedetomidine was administered, and his violent behavior and delirium significantly improved. The two episodes of hyperactive delirium following IV levetiracetam administration occurred at ages 3 and 5, resulting in extensive work up including laboratory testing and cranial imaging, along with cerebral spinal fluid analysis and were normal. IV dexmedetomidine provided rapid symptom relief to prevent harm for the child, staff, and family on both occasions.

## 1. Introduction

Severe hyperactive delirium is an incredibly rare side effect of levetiracetam and can result in hallucinations and self-harm behavior [1–3]. Treatment of severe hyperactive delirium can be challenging in pediatric patients. When environmental strategies fail, antipsychotics are sometimes used, but these may not be effective and can have severe side effects [4]. Dexmedetomidine, an alpha 2 agonist, has been used to manage symptoms of delirium in pediatric patients and is gaining popularity in treatment for pediatric patients with severe anticholinergic toxidrome [5, 6]. There is currently a paucity of information on using dexmedetomidine for levetiracetam-induced acute psychosis.

## 2. Case Presentation

### 2.1. Patient Information

A 5-year-old male presents to the emergency department (ED) following a seizure at home. The child was found in bed with his head turned to the right, arms and legs extended, and rhythmically shaking. The event lasted about one minute. He was evaluated in the ED after the seizure, and he was alert and interacting. Additional symptoms include two weeks of cough and congestion, fever (38.7°C) two days ago along with nausea, vomiting, and diarrhea for the past 24 hours. He had been receiving acetaminophen and ibuprofen for fever and abdominal pain for the past 48 hours. Two hours after the first seizure, he developed another brief seizure while in the ED, and he was given intravenous (IV) lorazepam 1 mg and IV levetiracetam 40 mg/kg. He was transferred to the pediatric inpatient ward where he had a third brief seizure three hours later. He was loaded with fosphenytoin 20 mg/kg IV. Postictal, he became very emotional, inconsolable, and combative. He was transferred to the pediatric intensive care unit (PICU) for further management.

His past medical history included a Lamin A/C gene (LMNA) mutation which also affected several family members including his father. This condition is associated with dilated cardiomyopathy with conduction disorders and arrhythmias which can develop at an early age [7]. The child had been followed every 6 months with pediatric cardiology and had a normal echocardiogram 2 months prior to this admission. The child was previously hospitalized for 4 days at age 3 when he presented in status epilepticus following a gastroenteritis illness. Following resolution of his seizures while in the hospital, he developed severe hyperactive delirium and was very combative. His work up during that time included a sedated MRI of his brain (normal), cerebral spinal fluid analysis (normal), and chemistry panel (normal) along with an EEG which showed no epileptiform abnormalities. He had persistent hyperactive delirium and was managed in the ICU with continuous dexmedetomidine infusion for 24 hours before his symptoms improved. Initially, he was started on levetiracetam in the ED and continued after discharge. His family reported ongoing behavior issues and levetiracetam was discontinued 2 weeks later. He was transitioned to oxcarbazepine and his behavior issues resolved. He remained seizure free for 1 year and was taken off oxcarbazepine at age 4.

**Figure 1:**
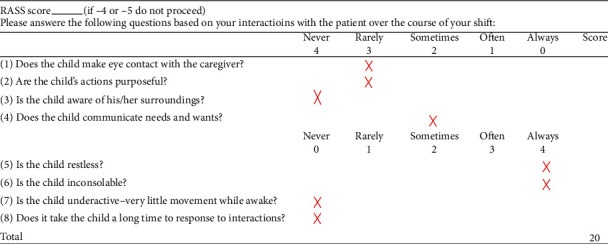
Cornell Assessment of Pediatric Delirium (CAPD).

Vital signs on admission to the PICU included oral temperature of 37.3°C, pulse rate 143 beats per minute, respiratory rate 24 breaths per minute, blood pressure 97/58 mmHg, and oxygen saturation 100% on room air. Patient weight is 22.7 kg. On examination, he had extreme agitation and was exceedingly difficult to console. He had a Pediatric Glasgow Coma Scale of 13 to 14 (E: 4, V: 3-4, and M: 6). He thrashed around the bed and was kicking and striking his family and staff. He was yelling and crying as he was focused on going to a water park that the family had planned the following week. His Richmond Agitation-Sedation Scale Score ranged from 3+ to 4+, and his Cornell Assessment of Pediatric Delirium (CAPD) score was 20 indicating delirium (see Figure 1). His pupils were symmetric and equal to light with intact intraocular motion. His abdomen was not distended and there was no abdominal pain with palpation. He had good strength and tone in all four extremities. Laboratory studies were unremarkable (see Table 1). An electroencephalogram (EEG) could not be completed due to the patient's agitation.

To reduce stimulation and allow the child to rest, environmental modifications included a quiet room with alarms and monitors turned off. He was given melatonin 3 mg and then diphenhydramine 12.5 mg which the family had given previously given at home to help him sleep. While in the PICU, he would rest for 1-2 hours and then wake up and was confused and disoriented. He continued to have agitation and combative behavior for the next 5 hours after these interventions. He was given risperidone 5 mg by mouth. For the next 2 hours, he continued to climb out of the bed, and when being held, he would strike his father. The family became exhausted from trying to console him. Through shared decision-making with the ICU team, it was decided to start him on dexmedetomidine. A dexmedetomidine 2 mcg/kg bolus was given over 1 hour followed by continuous infusion ranging from 0.2 to 0.5 mcg/kg/hr. He became drowsy and fell asleep for 1 hour before waking up and becoming more agitated. Dexmedetomidine was titrated up to 2 mcg/kg/hr and he was able to rest safely in his bed. His heart rate range decreased while on dexmedetomidine from 100 to 80 beats per minute, but he maintained adequate blood pressures with the lowest measured 89/54 mmHg. Once he fell asleep, an EEG was obtained which was negative for epileptiform abnormalities or focal changes, and the background was most consistent with sleep state. Given the persistence in his agitation and delirium along with his recent illness with fever and seizures, a diffusion-weighted MRI brain with and without contrast was obtained, which revealed nonspecific minimal FLAIR bright signal in the subparietal white matter with uncertain clinical significance. A lumbar puncture was obtained while on the continuous infusion of dexmedetomidine which he tolerated well without any complications. The cerebral spinal fluid (CSF) analysis revealed WBC 0/mm^3^, RBC 1/mm^3^, glucose 64 mg/dL, protein 16 mg/dL, enterovirus CSF-PCR negative, HSV 1, and 2 DNA QL PCR negative. CSF culture showed no growth for 5 days.

Eight hours after starting the dexmedetomidine infusion, he woke up and was calmer. He began asking more appropriate questions and his delirium had improved. Dexmedetomidine was discontinued. He was able to play in his room, throw a ball, and catch at the bedside the following morning but had a mildly ataxic gait. The child was discharged home 8 hours after discontinuing dexmedetomidine continuous infusion as he had significantly improved. Oxcarbazepine 300 mg BID was restarted for seizure maintenance therapy under the guidance and follow-up with pediatric neurology.

Given the strong association with this child receiving levetiracetam IV and developing severe hyperactive delirium on two separate occasions, levetiracetam was added to the child's allergy warning list to prevent future administration. A follow-up phone call was made to the family 4 weeks after discharge. He remained seizure-free and was doing well. The mild ataxia had resolved within two days, and no residual behavioral changes were seen. The family did not feel that further psychiatric evaluation was indicated following discharge as his symptoms had significantly improved. They were instructed to monitor for changes in behavior and notify their primary care provider and neurologist if they observed any issues.

## 3. Discussion

Over the past several years, we have observed an increase in the utilization of IV levetiracetam as a second-line agent for status epilepticus in pediatric patients treated in EDs who are transferred to our PICU for further management. Levetiracetam is as successful for seizure cessation as alternative second line agents, such as fosphenytoin and valproic acid, but associated with less severe side effects [8]. Levetiracetam's adverse effect profile is also significantly improved compared to alternative agents phenytoin and phenobarbital [9, 10]. Phenobarbital's association with CNS and respiratory depression deters use and incorrect preparations of phenytoin have been associated with death [11]. Another benefit of levetiracetam over other agents is that it can be administered over a shorter period, potentially decreasing time to seizure cessation [12]. Levetiracetam has multiple suspected mechanisms of action on the nervous system for cessation of status epilepticus from binding the Synaptic Vesicle Protein 2A protein (SV2A) which include the inhibition of calcium release from intraneuronal stores, and the neuromodulation of gamma aminobutyric acid (GABA) receptors [13, 14]. Behavioral side effects from levetiracetam reported in children include agitation (3.4%), mood swings (2.1%), aggression (8.2%), and abnormal behavior (5.6%) [15]. The mechanism responsible for the behavioral side effects of levetiracetam such as acute severe hyperactive delirium is unknown, but evidence suggests that a negative modulating effect on *α*-amino-3-hydroxy-5-methyl-4-isoxazolepropionic acid (AMPA) receptors may contribute to increased aggressive behavior [15]. Our patient had a previous reaction to levetiracetam at 3 years of age causing hyperactive delirium. He improved over several days, but he continued to have significant behavioral issues such as aggression at home. Levetiracetam was discontinued and his behavior improved. When he presented 2 years later in status epilepticus, he was given levetiracetam in the ED. The status epilepticus resolved, but he developed severe hyperactive delirium. The behavioral effects of levetiracetam may be multifactorial, and influences such as epigenetics, genetic regulation of neurotransmitters (GABA, glutamate), synaptic channels with effect on AMPA receptors, serotonin (5-HT) modulation, or even intracellular events following a seizure could play a role in the behavioral side effects observed [15]. The seizure medication brivaracetam, which also has a high affinity for SV2A, does not have activity on other neurotransmitters such as 5-HT, GABA, or AMPA receptors compared to levetiracetam and is associated with less behavioral side effects [16].

Levetiracetam does appear to have an indirect agonist effect on alpha 2 adrenergic receptors in the peripheral nervous system resulting in an antinociceptive effect but is not well understood [14, 17]. The effect on the central nervous system has not been investigated. Alpha 2 adrenergic receptors have an inhibitory effect on the sympathetic nervous system [18]. With the administration of dexmedetomidine, an alpha 2 receptor agonist medication, we observed a significant improvement in the patient's symptoms of acute hyperactive delirium and aggressive behavior induced by levetiracetam administration on two separate occasions [19].

Dexmedetomidine has been used to treat anticholinergic toxidrome, emergence delirium and is an effective sedative used in the PICU [20]. The downstream central nervous system effect of alpha 2 receptor stimulation results in inhibition of adenylyl cyclase which causes hyperpolarization of noradrenergic neurons in the locus ceruleus. This leads to potassium efflux through calcium-activated channels preventing calcium ions from entering the nerve terminal which suppresses neural firing and inhibits norepinephrine release/reducing ascending noradrenergic pathways [19]. Dexmedetomidine is hepatically metabolized with a half-life ranging from 2-3 hours. Loading doses range from 0.25 to 6 mcg/kg/hr and maintenance doses typically from 0.2 to 1.4 mcg/kg/hr [20]. We observed immediate improvement in his hyperactive delirium symptoms when we used 2 mcg/kg/hr. The most common side effects of dexmedetomidine include hypotension and bradycardia through stimulation of the alpha 2A-AR receptors on the heart and peripheral vasculature, while hypertension can also occur through stimulation of the alpha 2B-AR receptor causing vasoconstriction [21]. Our patient did not have any of these side effects. Dexmedetomidine has been shown to safely improve symptoms of severe hyperactive delirium in adult ICU patients who are refractory to haloperidol [22], and recent literature indicates its role in prevention and treatment of delirium [22, 23].

Pediatric patients with severe hyperactive delirium present diagnostic and management challenges. Initial efforts focus on preventing the patient from harming themselves, family, and staff, along with identifying triggers. Medical conditions such as hypoxia, infection (meningitis/encephalitis), new organ dysfunction, renal failure, metabolic disturbances (hyponatremia/hypoglycemia, hypercalcemia), medications (anticholinergics, benzodiazepines), and severe pain need to be considered as causes for severe hyperactive delirium in the pediatric patient [24]. Environmental contributors such as restraints, noise, strangers, lights, absent family members) may also contribute to delirium. Multiple environmental modifications were pursued for our patient along with administration of risperidone. The child remained very agitated and combative, striking family and staff. Continuous infusion of high-dose dexmedetomidine immediately improved symptoms.

From our review, we describe the first case of a child on two separate occasions, developed acute severe hyperactive delirium with aggressive harmful behavior following the administration of IV levetiracetam and had significant improvement in symptoms with high-dose intravenous dexmedetomidine, with increased alertness within 12 hours and discharge on subsequent day. As compared to a previous report of a 12-year-old girl developing acute psychosis 10 days after the initiation of levetiracetam, our patient developed severe symptoms within 6 hours of levetiracetam administration on two separate occasions [3]. Previous case reports discuss using dexmedetomidine in the treatment of anticholinergic toxidrome which can be incredibly challenging to manage in the ICU. Behavioral changes from levetiracetam do occur but severe symptoms are infrequent, and the exact mechanism is unknown. Discontinuation of levetiracetam was done in this child's case for the second time and he was started back on oxcarbazepine for seizure maintenance therapy. We propose that dexmedetomidine may be considered to safely treat symptoms of levetiracetam induced severe hyperactive delirium in pediatric patients.

## 4. Conclusion

We describe a child who received IV levetiracetam on two separate occasions both of which resulted in aggressively combative and harmful behavior towards family and staff. Levetiracetam-induced hyperactive delirium with aggression in a young child is an exceedingly rare side effect and can be particularly challenging to manage. Continuous infusion dexmedetomidine at high doses may be effective at treating acute hyperactive delirium symptoms associated with this condition and prevent patient self-harm along with harming family members and the healthcare team.

## Figures and Tables

**Table 1:** Laboratory test results.

*Complete blood count*	
White blood cell count	6.9 K/*μ*L
Hematocrit	35.5%
Platelets	258 K/*μ*L
Lymphocytes	23%
*Comprehensive metabolic panel*	
Sodium	134 mmol/L
Potassium	3.8 mmol/L
Glucose	102 mg/dl
Blood urea nitrogen	15 mg/dl
Creatinine	<0.5 mg/dl
Aspartate transaminase (AST)	83 IU/L
Alanine transaminase (ALT)	69 IU/L
*Infection screening*	
Influenza A and B PCR	Negative
SARS CoV2 PCR	Negative
Throat culture (alpha beta hemolytic Streptococcus)	Negative
*Cerebral spinal fluid*	
White blood cells	0/mm^3^
Red blood cells	1/mm^3^
Glucose	64 mg/dL
Protein	16 mg/dL
Culture	Negative

## Data Availability

No data were used to support this study.
